# Micro/Nano-pore Network Analysis of Gas Flow in Shale Matrix

**DOI:** 10.1038/srep13501

**Published:** 2015-08-27

**Authors:** Pengwei Zhang, Liming Hu, Jay N. Meegoda, Shengyan Gao

**Affiliations:** 1 State Key Laboratory of Hydro-Science and Engineering, Department of Hydraulic Engineering, Tsinghua University, Beijing 100084, P. R. China; 2 Department of Civil and Environmental Engineering, New Jersey Institute of Technology, Newark, NJ 07102, USA

## Abstract

The gas flow in shale matrix is of great research interests for optimized shale gas extraction. The gas flow in the nano-scale pore may fall in flow regimes such as viscous flow, slip flow and Knudsen diffusion. A 3-dimensional nano-scale pore network model was developed to simulate dynamic gas flow, and to describe the transient properties of flow regimes. The proposed pore network model accounts for the various size distributions and low connectivity of shale pores. The pore size, pore throat size and coordination number obey normal distribution, and the average values can be obtained from shale reservoir data. The gas flow regimes were simulated using an extracted pore network backbone. The numerical results show that apparent permeability is strongly dependent on pore pressure in the reservoir and pore throat size, which is overestimated by low-pressure laboratory tests. With the decrease of reservoir pressure, viscous flow is weakening, then slip flow and Knudsen diffusion are gradually becoming dominant flow regimes. The fingering phenomenon can be predicted by micro/nano-pore network for gas flow, which provides an effective way to capture heterogeneity of shale gas reservoir.

According to the U.S. Energy Information Administration (EIA) data, as of 2012 shale gas is more than 40% of U.S. domestic total natural gas production and it is expected to reach 50% in 2039. The Chinese government is also determined to exploit shale gas resources and is planning to reach an annual production rate of 60 to 100 billion cubic meters by 2020^1^. Hence it is essential to understand shale reservoir properties and gas flow mechanisms in order to achieve above shale gas production goals.

Shale is an ultra-tight rock with relatively low pore connectivity^2^,^3^, and extremely low permeability of 10^−18^ to 10^−21^ m^2^ 4,5. Hydraulic fracturing breaks up shale matrix and connects natural fractures to create flow paths to improve the reservoir connectivity. Thereafter, free gas can quickly release along hydraulic fractures, and gas stored in the matrix slowly transport to connected fractures^6^,^7^. Finally, the adsorbed gas in pore surfaces begins to desorb due to reduction in pressure gradient. Therefore, gas storage in the shale matrix and matrix gas flow pattern are important for prediction of gas production.

Pore size of shale matrix ranges from several nanometers to several hundred nanometers^7^,^8^,^9^,^10^,^11^,^12^, and pore throat size is even much smaller^9^,^13^, which is closer to the mean free path of gas molecule. Therefore, gas flow in such nano-scale channels is not governed by viscous flow, where the drag force along rough pore surface and gas molecular collision with pore wall become non-ignorable. Knudsen number (*K*_n_) is defined to separate different dominant flow regimes, and expressed as 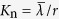 , which is the ratio between gas molecular mean free path 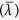 and gas flow characteristic size (*r*) in porous media^14^,^15^,^16^,^17^,^18^. The Knudsen number accounts for the frequency of molecule-molecule and molecule-wall collisions based on gas molecular dynamics theory^19^,^20^, which also can be used to separate different flow regimes. For very small values of Knudsen number (*K*_n_ < 0.01), the characteristic size of gas flow channels is relatively larger than the mean free path of gas molecule, which enables gas flow in continuous state and viscous flow type is available. As the Knudsen number becomes larger (0.01 < *K*_n_ < 0.1), no-flow assumption near pore surface becomes invalid, and slipping along pore surface occurs^21^,^22^. Thereafter, in the transition flow regime (0.1 < *K*_n_ < 10) gas slippage along pore surface and collision with pore wall simultaneously occur^23^. When *K*_n_ > 10, gas flow regime becomes free molecular flow, in which gas molecule-molecule interaction turns weak, and molecule-wall interaction is intensified. In real shale reservoir gas flow, flow regime changes with pore gas pressure, and hence a single flow regime cannot accurately predict mass flux and pressure distributions. Therefore, a theoretical model to capture dynamic gas flow under various flow regimes would be of valuable, and has attracted interests of many researchers. Beskok and Karniadakis (1999) developed a unified gas flow model by defining a rarefaction factor, which explained flow regime changes with variation in gas molecular interaction frequency. Florence *et al.* (2007) based on large volume of industrial data, defined a pseudo Knudsen number and modified the model proposed by Beskok and Karniadakis (1990), which provided refinements to previous models, especially for ultra-tight porous media^24^. Additionally, Civan (2010) proposed a simple inverse power-law relationship of rarefaction factor and correlated with experiment data. These unified models were obtained from straight tube and the validity for nano-scale shale matrix is uncertain. The flow regimes in typical shale reservoir are mainly slip and transition flows^25^,^26^,^27^,^28^, hence the gas flow model for shale matrix should combine slip flow and Knudsen diffusion^23^,^26^,^29^,^30^. A true molecular simulation using Molecular Dynamics (MD) or Lattice Boltzmann Method (LBM) would be a preferred approach. Chen *et al.* (2015) simulated the three-dimensional (3D) nanoscale porous structures of shale using a reconstruction method called Markov chain Monte Carlo (MCMC) based on SEM images of shale samples. Then they used LBM to simulate nanoscale shale gas flow. The method proposed by Chen *et al.* (2015) is quite fundamental but computationally very intensive, hence a fairly accurate but simpler method using typical shale reservoir data is proposed in this research.

The pore-scale simulation is an efficient way to understand micro/nano scale fluid flow^31^,^32^,^33^,^34^,^35^,^36^,^37^,^38^,^39^,^40^,^41^,^42^. The shale matrix is porous media of low connectivity and it is time consuming to perform permeability tests. Hence pore network models may provide an effective way to obtain basic hydraulic parameters for shale reservoir, to understand dynamic migration of shale gas and to predict reservoir gas production. The pore size distribution in shale matrix has bimodal characteristics^10^,^43^. Mehmani *et al.* (2013) established a multi-scale pore-network with a constant coordination number of 4 by extracting pore-network from a dense random pack of spheres by Delaunay tessellation method, and the results show that gas flow in shale matrix is mainly determined by nano-pore fraction.

In this study, a mathematical micro/nano-pore network model is proposed, where pore sizes, pore throat sizes and coordination numbers satisfy typical shale reservoir data. Isolated pores and clusters also exist in this pore network, while the connected backbone is the research focus of this study. Furthermore, the pore-network model has to satisfy the law of statistics, which is verified in a numerical simulation. Based on the proposed pore network, numerical experiments of apparent permeability tests were conducted for two types of pore throat distributions, i.e., a constant pore throat size and that obeys normal distribution. Then the dynamic gas migration after considering Klinkenberg slip effect and Knudsen diffusion is analyzed. The slip flow and diffusion theories coexist in the shale matrix pore network as the flow regimes range from slip to transition flow^23^,^25^. In this work, the two flow regimes for gas mass flux contribution at different stages are considered and dynamic gas flow is analyzed. Finally, the fingering effect in the pore-scale network model is incorporated, hence the scaled up pore network model can effectively capture the heterogeneity of the porous media.

## Results

### Structure of shale matrix pore-network model

**Figure 1:**
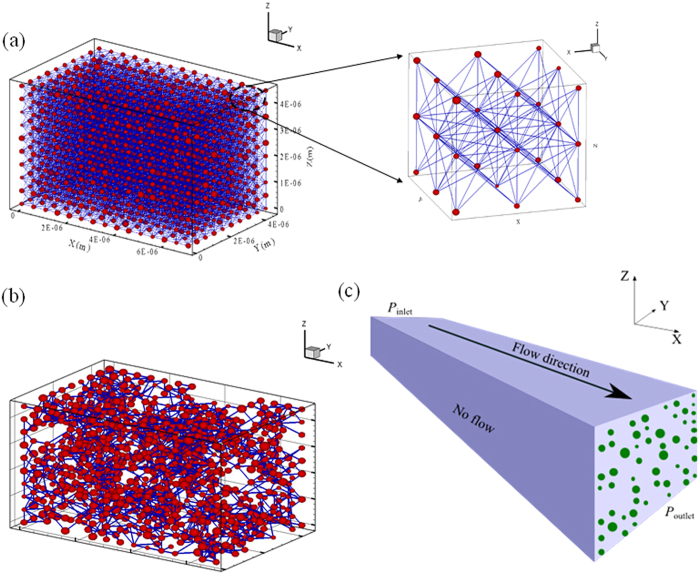
Micro-pore network model for gas shale matrix. (**a**) Fully connected pore network; (**b**) Extract backbone; (**c**) Sketch of pores in shale matrix based on the extracted backbone shown in (**b**).

Figure 1(a) presents a generated fully-connected micro/nano pore network. In order to match the measured data, the interconnected coordination bonds were eliminated at inlets and outlets to avoid planar flow. A dilution procedure was developed to reflect the low connectivity of shale matrix, and the backbone of the pore-network after the isolated pores and isolated pore clusters were eliminated as shown in Fig. 1(b). The gas flow is along X direction as shown in Fig. 1(c). Pressure difference was applied to the inlet and outlet boundary surface. A constant pressure (1 MPa) was maintained at the outlet boundary during shale gas exploitation. The other boundary surfaces are set no flow conditions. In this research, the pore size distribution can be divided into two parts: large pores and small pore throats. Both large pores and small pore throats obey normal distribution, where the average pore size is 300 nm and average pore throat size is 6 nm^13^. The pore diameter ranges from 100 nm to 500 nm, and pore throat size ranges from 1 nm to 10 nm. As reported in many literatures^44^,^45^,^46^, the average coordination number for sandstones is approximately 4. The coordination number decreases with decreases in porosity, thus average coordination number is assumed as equal to 3 for shale matrix in the proposed pore network model is reasonable, and it also obeys normal distribution. The upper bound of coordination number is 26 as each pore can be connected to 26 adjoining pores for gas transport^47^,^48^.

### Theoretical gas flow model of shale matrix

**Figure 2:**
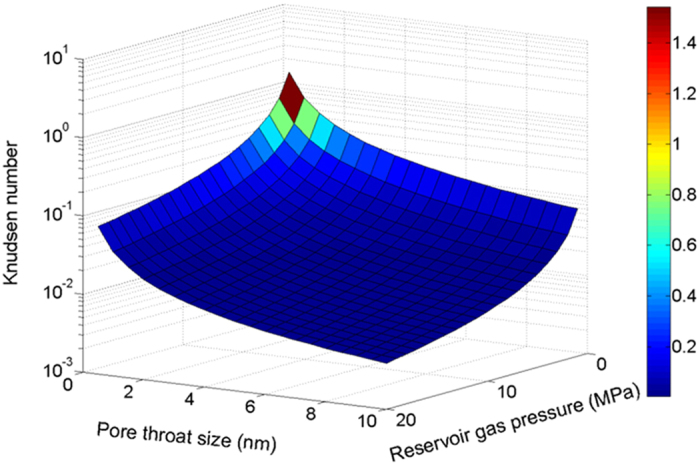
Knudsen number variation according to typical shale reservoir data.

During the dynamic gas flow, the variation in Knudsen number results in the change of flow regimes. Figure 2 illustrates the relationship between the Knudsen number and the pore throat size as well as reservoir gas pressure. The Pore throats were used in this work instead of larger pores for gas flow computation in shale matrix. When the pore throat sizes are used for the flow computation the Knudsen number ranges from 0.01 to 1, and the Knudsen number may larger than 1 when the reservoir pressure decreases. Therefore, the typical gas flow in shale matrix is mainly slip and transition flow. Hence it is necessary to combine the slippage effect and Knudsen diffusion in the theoretical model^23^.

The single fluid phase flow rate in a pipe can be described by Hagen-Poiseuille equation, if it is applied to gas phase considering gas compressibility, the mass flux equation is written as:


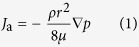


where *ρ* is the gas density equals *pM*/*RT*, *p* is the reservoir gas pressure in Pa, *M* is gas molar mass in kg/mol, *R* is universal gas constant in J/mol/K, *T* is the thermodynamic temperature in K, *μ* is the gas dynamic viscosity in Pa s.

For slippage effect, the gas slip along pore wall was considered by proposing a correction factor (*F*)^22^,


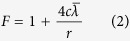


where *c* is a collision proportionality factor, normally given as equal to 1.0, 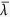 is the gas molecular mean free path, which can be obtained by the gas kinetic theory:


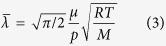


Then the modified gas mass flux is


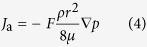


The diffusion part can be described by the Knudsen diffusion model^23^:


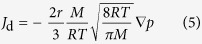


**Figure 3:**
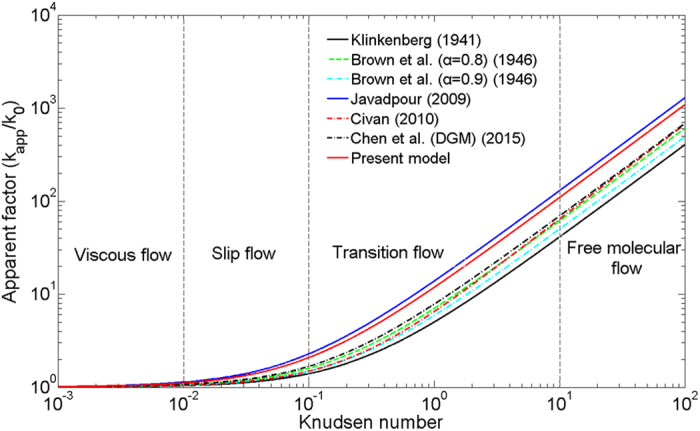
Comparisons of apparent permeability model (“α’’ is the tangential momentum accommodation coefficient in Brown’s model, reflect wall roughness).

Combining Eq. (4) and (5) to obtain:


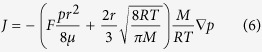


where *J* is the mass flux in kg/m^2^/s. Eq. (6) was used to calculate the discharge through each coordination bond connecting two adjacent pores. It is in essence similar to Javadpour (2009) flow rate equation, except a minor difference in the slip part as shown in Fig. 3.

Figure 3 shows several typical apparent model simulation results. As it indicates, apparent permeability is higher than intrinsic permeability, especially for higher Knudsen numbers. The apparent permeability models of Florence *et al.* (2007) and Civan (2010) are multi-flow regimes, which are obviously higher than results of Klinkenberg (1941). The model proposed by Brown *et al.* (1946) is similar to Klinkenberg (1941), while the former considered the wall roughness by bringing in the tangential momentum accommodation coefficient. When the tangential momentum accommodation coefficient (α) increases the apparent permeability decreases. Specifically, when α approaches 1, the two apparent permeability models are the same. Additionally, when α approaches 0.8 the Brown (1946) model is very close to those of Florence *et al.* (2007) and Civan (2010) even in transition flow region. This indicates that Brown (1946) model can account for the gas molecular interactions with pore walls. Therefore, in present mass flux model Klinkenberg (1941) slip flow combined with Knudsen diffusion (Eq. (6)) was applied in the transient flow region, which may accurately account the contribution from the two regimes separately. Javadpour (2009) studied the gas flow regimes in nanoscale shale matrix, and the Brown’s slippage effect and Knudsen diffusion were considered in the apparent permeability model. The apparent permeability model proposed by Chen *et al.* (2015) was based on the Dusty gas model (DGM), which considered the viscous flow and Knudsen diffusion.

### Variation of Dominant flow regimes

**Figure 4:**
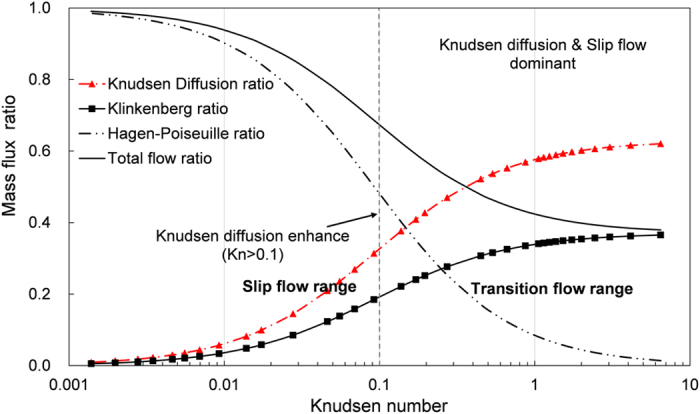
Dynamic mass flux ratio by different flow regimes with the Knudsen number variation.

In shale reservoir, free gas release quickly from micro fracture and connected shale matrix after hydraculic fracturing. With the dcrease of reservoir gas pressure, the dominant flow regimes for gas exploitation varied at each stage. Based on gas kinetic theory, the variation of macro flow regimes is due to the interaction of gas molecules with themselves and with the wall. At each stage, the flow regimes include viscous flow, slip flow and Knudsen diffusion, and they are mixed with differen proportions as shown in Fig. 4. During the viscous flow range and slip flow range, Knudsen diffusion is almost negligible and the pressure driven flow is the main gas production source. However, the mass flux ratio contributed by typical Hagen-Poiseuille viscous flow model gradually decreases with the increase of Knudsen number, especially in the transition flow range and the viscous pipe flow almost extinct. In contrast, Klinkenberg slippage effect and Knudsen diffusion tend to play a major role in transiton flow range. Specifically, total flow in Fig. 4 includes Hagen-Poiseuille flow and Klinkenberg slip flow, and the latter takes up almost 40% of total flux when Knudsen number is larger than 1. The Knudsen diffusion starts to enhance sgnificantly when the Knudsen number is larger than 0.1, and it contributes to more than 60% of total mass flux at the later stage of transition flow range. Therefore, at the later stage of gas exploitation, gas production is mainly due to Knudsen diffusion and slip flow.

### Dynamic gas flow in pore-network model

Gas flow through the pore network is a dynamic process and each pore satisfies conservation of mass equation. Take pore *i* for example, it connects with several adjacent pores. During each time step, pore throat pressure is equal to the average pressure of connected two pores. From the time step *k* to *k* + 1, the mass changes in pore *i* can be balanced by the summation of all the mass flux related to pore *i*:


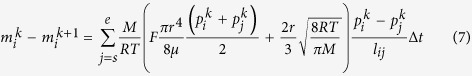


For ideal gas, the gas mass store in pore *i* at *k* time step is:


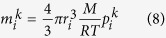


Substitute Eq. (8) into Eq. (7) yields:


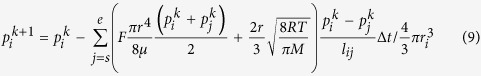


Eq. (9) shows the iteration process for pressure. At the start, pressure difference only applied to the boundary pores and pores connected to it, subsequently it will dynamically cover all pores in the pore network. During this computation, time step is crucial for convergence. The Courant number which reflects the relation between time step and space step was used to verify the validity of numerical simulation. Courant number should smaller than 1 to ensure the computational stability, thus the range of time step can be determined by Eq. (10).


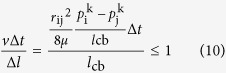


where the *l*_cb_ is the coordination bond, which connects, pore *i* and pore *j* in the pore network.

**Figure 5:**
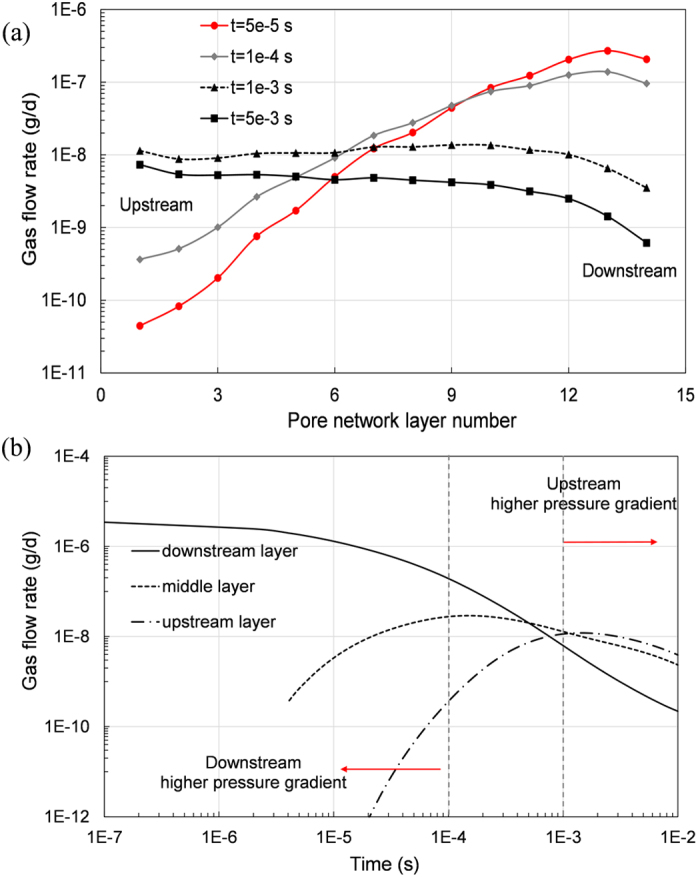
Dynamic gas flow rate. (**a**) flow rate of each layer at certain time; (**b**) flow rate changes with time of three typical pore network layers.

The advantage of pore network simulation is that the dynamic gas transfer can be captured. Figure 5(a) presents the variation of gas flow rate of whole pore network. At the time of hydraulic fracture, gas flow rate near the outlet or dowmstream fracture is higher because of the higher initial pressure gradient. With the passage of time after hyraulic fracture, the gas flow rate in the inner layer shows an ascending tendency. After relatively balanced gas flow rate is reached, like the time node of 1 ms, gas rate of all layers of the pore network gradually decrease. In order to explore the relation of gas flow rate changes of three typical pore network layers (downstream, middle and upstream layers) are analyzed in Fig. 5(b). The pressure gradient for downstream layer is continously decreasing, which leads to steadily decreasing gas flow rate during gas exploriation. While the gas flow rate for middle and upstream layers experience an ascending rates initially followed by a declining rates. This observation can be explained using the initial condition of the reservoir, which is constant pressure for all pores, therefore, the initial pressure gradient for inner layer is zero, and it experiences increase initially and then decrease later. Additionally, at the end of gas exploriation the larger pressure gradient isoline moves into inner layers, as shown in Fig. 5(b).

## Discussion

### Apparent permeability in shale matrix

**Figure 6:**
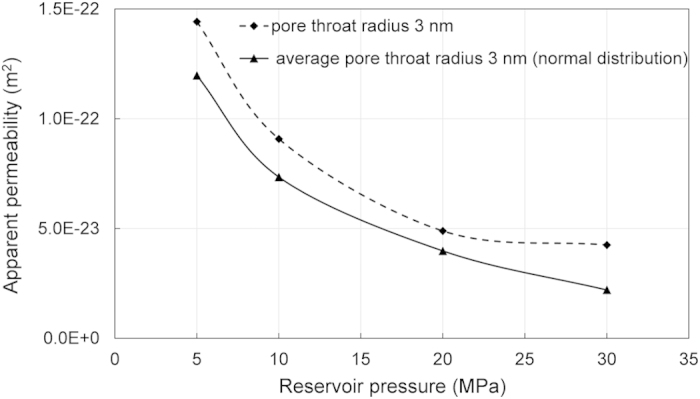
Sensitivity analysis of apparent permeability to pore throat size and reservoir gas pressure.

Figure 6 shows the apparent gas permeability of shale matrix using the proposed pore network, where two types of pore throat size distribution at different reservoir pressure are compared. Scenario 1 is for constant pore throat size, and Scenario 2 is for pore throat size obeying normal distribution. The results indicate that apparent permeability is sensitive to reservoir gas pressure and pore throat size. The gas density increases if reservoir gas pressure increases, which leads to decrease in kinematic viscosity (dynamic viscosity divides gas density) and decrease in apparent permeability. As it shows, the apparent permeability of constant pore throat size model is larger than that for the normally distributed one. Although the apparent permeability for Scenario 1 is approximately 2 times larger than Scenario 2, it still indicates that apparent permeability value of shale depends on the proportion small pore throat. Additionally, the results also show that the apparent permeability obtained from laboratory tests under lower pressures is higher than the permeability values of the real reservoir.

### Gas flow rate using different theoretical models

**Figure 7:**
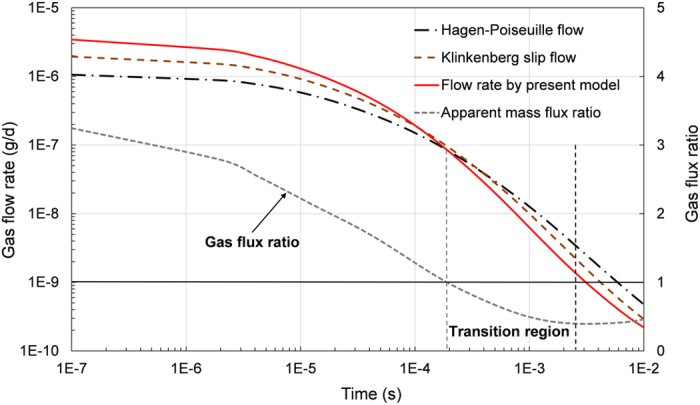
Comparisions of gas flow rate by different theorectical flow models.

Figure 7 compares the gas flow rates from three theoretical models (Hagen-Poiseuille flow, Klinkenberg slip flow, and flow by present model) using the proposed pore network model, the results show that the gas flow rate of proposed model considering slip flow and Knudsen diffusion is significantly higher than viscous flow model and Klinkenberg slip flow at the early time. However, according to the initial and boundary conditions for the pore network, the stored mass of gas in the shale matrix is assumed and hence the dynamic gas flow during gas extraction can be divided into three stages. At the initial stage, the reservoir gas pressure is relatively high and the pressure gradient decreases rapidly (larger slope) for the proposed model than that for other two models. Thus the gas flux ratio, defines as the flux calculated from the proposed model to that of viscous model, is gradually decreases from a starting value of slightly over 3.2. In the middle stage, with decreases of pore gas pressure in the proposed micro/nano network model, there will be a transition region where the gas flux calculated from the proposed model is less than that from other models (gas flux ratio is less than 1). In the final stage, the slope of gas flux ratio at the transition region is gradually decreasing, and at the last period of gas exploitation the low gas pressure leads to a higher Knudsen number, therefore the slip flow and Knudsen diffusion dominates and hence the gas flow ratio begins to increase (the ratio tends to increase slightly).

### Fingering effect

**Figure 8:**
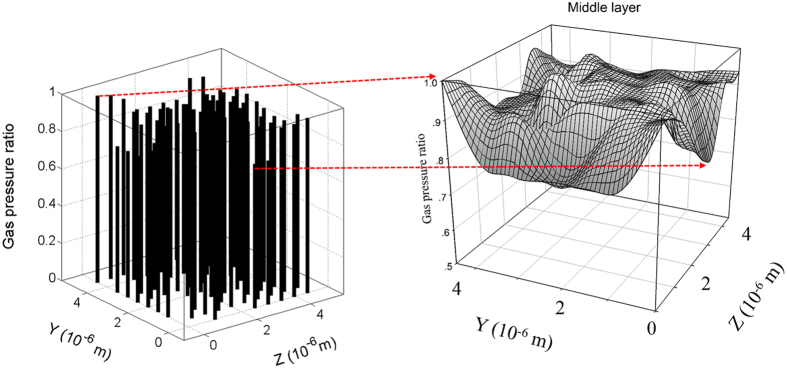
The fingering effect of gas flow in shale matrix pore network.

Heterogeneity is quite common in rock and soil. Macro simulation of heterogeneity in hydrogeology researches often assumes the hydraulic conductivity satisfy certain distribution^49^,^50^. Unfortunately, the hydraulic conductivity of *in-situ* shale reservoir is too difficult to measure due to deep burial conditions and extremely low permeability values. The Micro/Nano pore network analysis of gas migration in shale matrix may provide insights to the study the heterogeneity. Figure 8 shows a pressure distribution of the same pore network layer where the pore size distribution is according to the real shale matrix pore size range and the gas pressure ratio (gas pressure of pore *i* divides maximum pore pressure of this layer) is a curved surface demonstrating the fingering effect. It means that gas flow occurs through least resistant paths corresponding to larger pore throats in this pore network model of the shale matrix. In the proposed pore-network model, the micro fractures are not considered and the model size is only several microns. The up-scaled pore network model considering micro fracture should be able to accurately account for the fingering effect of preferential flow in real shale reservoir gas flow. However, it is out of the scope of this work.

The pore-scale simulation in shale matrix is an attractive way to study gas flow and predict long-term gas production. The impact of isolated pores in the pore network is not considered in this paper. Furthermore, with the gas release, the effective stress of shale reservoir increases, and this may lead to compression of reservoir and change of the permeability of shale matrix, which is also not considered in this paper. Hence it is quite promising to further develop the pore network model to perform more realistic simulations.

## Methods

### Porosity of the pore-network model

The methodology to generate pore network for typical porous media has been clearly presented^46^,^47^,^48^, however the low connectivity is the key characteristic of shale matrix, which should be adequately considered during the construction of pore structure. As mentioned before, the pore size, porosity and pore connectivity are very small for gas shale matrix. These parameters are essential for micro/nano-pore network analysis.

According to data from Barnett, Marcellus, Haynesville, and Eagle Ford shale, the shale porosity ranges from 1% to 10%^7^,^8^,^10^,^51^. To generate the regular micro/nano-pore network, the initial porosity was assumed to be 7% for the fully connected network, which is consistent with data for the Marcellus shale^7^. For a regular pore network, the pore spacing (pore center to center distance) is constant and determined by porosity. First, the number of pores in each direction is assumed in this model (*n*_x_ = 15, *n*_y_ = 10, *n*_z_ = 10), which should satisfy representative property of gas flow^32^,^40^,^48^. Then, the volume of all pores is calculated, and with the porosity the volume of the model *V* is obtained. Finally, the boundary length in each direction can be obtained using model volume *V* (*l*_x_*l_y_*l_z_ = *V*), and pore spacing by dividing length by number of pore numbers in each direction. Although pore center distance is constant for the whole model, the coordination bond length is varied by connecting to different pores where the coordination bond length (*l*_cb_) is equal to pore center distance minus the radius of the adjacent two pores.


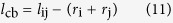


where *l*_ij_ is the pore center distance between pores *i* and *j* in m, *r*_i_ and *r*_j_ are the radius of pores *i* and *j* respectively in m. When two adjacent pores are interconnected, which means there is no coordination bond, then the two pores are merged into one larger pore^47^.

### Pore connectivity

Narrow pore throat is disordered in shale matrix. Therefore, a multi-directional pore throats may capture this property. Raoof and Hassanizadeh (2009) accomplished this by assigning 26 coordination number for each nodes. The 26 coordination bonds were not suitable for low connective shale and dilution coordination bond is necessary. Gao *et al.* (2012b, a) proposed rigorous dilution procedure for dynamic two-phase flow in porous media. However, this dilution procedure is not suitable for shale matrix as shale matrix is a relatively low interconnected porous media. The dilution procedure in shale matrix is similar to that in percolation theory where each coordination bond has a probability threshold which determines if the bond is opened or blocked^48^,^52^. The probability threshold for bond connect pore *i* and pore *j* is as follows:


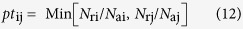


where *N*_ri_, *N*_rj_ are the random distribution coordination number for pores *i* and *j* respectively, *N*_ai_, *N*_aj_ are the assigned coordination number for pores *i* and *j* in the fully connected pore network. Similar to the term “elimination number” defined by Raoof and Hassanizadeh (2009), if the elimination number for a bond is larger than the corresponding probability threshold, then that bond will be eliminated. As average coordination number for shale matrix is relatively small, the probability threshold for each bond will be much smaller. Therefore, elimination number obeying random distribution between 0 and *η*, where *η* is the reduction factor ranging from 0 to 1 was used. The reduction factor depends on the connectivity of pore network and it is a function of average coordination number and porosity. If the connectivity is high, *η* can be set smaller, otherwise, there will be more isolated pores. Besides, *η* can be different in each direction, which makes our model quite flexible to simulate material anisotropy. The pore-network model in this work can also be simplified to a constant coordination number model^47^. For shale matrix pore network model, *η* value has to satisfy statistics law, which is verified in a numerical simulation. Figure 4(b) shows the interconnected pore network after dilution. For this average coordination number and porosity in shale matrix pore network, 0.45 is an upper bound value for reduction factor. The statistic value from 25 groups tests were conducted, and the coefficients of variation (total pores and pore coordination numbers) are almost within 5%, which shows a good stability.

### Permeability tests

Steady state flow is usually assumed in permeability tests of pore network models^3^,^47^, as it can establish mass balance equation 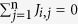 for each pore. Unfortunately, for shale matrix pore network in this work, with low connectivity directly solving the equation set will lead to a large sparse matrix which is difficult to invert. Furthermore, as it is nonlinear equation and the convergence for iteration method like Newton-Raphson is time consuming. Therefore, a transient permeability test method was applied, which set constant pressure difference between inlet and outlet, the gas flow will reach steady state and the permeability can be obtained at that state. The convergence criteria is defined as that searching for the maximum pressure difference during time step *k* and *k* + 1 for all pores, and let it be less than the allowable error: Max 
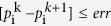
. The allowable error to reach steady state was set as: 
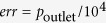
, it is 0.1 kPa and much less than the MPa range in reservoir gas pressure.

After the steady state was reached, the permeability for the whole shale matrix pore network can be calculated as follows:


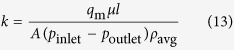


where *q*_m_ is the reservoir average gas flow rate in kg/s, *A* is the cross section of the pore network in m^2^, *l* is the length of the pore network along flow direction in m, *ρ*_avg_ is the average gas density through the pore network in kg/m^3^.

## Additional Information

**How to cite this article**: Zhang, P. *et al.* Micro/Nano-pore Network Analysis of Gas Flow in Shale Matrix. *Sci. Rep.* **5**, 13501; doi: 10.1038/srep13501 (2015).
